# A stomata classification and detection system in microscope images of maize cultivars

**DOI:** 10.1371/journal.pone.0258679

**Published:** 2021-10-25

**Authors:** Alexandre H. Aono, James S. Nagai, Gabriella da S. M. Dickel, Rafaela C. Marinho, Paulo E. A. M. de Oliveira, João P. Papa, Fabio A. Faria

**Affiliations:** 1 Instituto de Ciência e Tecnologia, Universidade Federal de São Paulo, São José dos Campos, São Paulo, Brazil; 2 Instituto de Biologia, Universidade Federal de Uberlândia, Uberlândia, Minas Gerais, Brazil; 3 Department of Computing, São Paulo State University, Bauru, São Paulo, Brazil; University Tunku Abdul Rahman, MALAYSIA

## Abstract

Plant stomata are essential structures (pores) that control the exchange of gases between plant leaves and the atmosphere, and also they influence plant adaptation to climate through photosynthesis and transpiration stream. Many works in literature aim for a better understanding of these structures and their role in the evolution process and the behavior of plants. Although stomata studies in dicots species have advanced considerably in the past years, even there is not much knowledge about the stomata of cereal grasses. Due to the high morphological variation of stomata traits intra- and inter-species, detecting and classifying stomata automatically becomes challenging. For this reason, in this work, we propose a new system for automatic stomata classification and detection in microscope images for maize cultivars based on transfer learning strategy of different deep convolution neural netwoks (DCNN). Our performed experiments show that our system achieves an approximated accuracy of 97.1% in identifying stomata regions using classifiers based on deep learning features, which figures out as a nearly perfect classification system. As the stomata are responsible for several plant functionalities, this work represents an important advance for maize research, providing an accurate system in replacing the current manual task of categorizing these pores on microscope images. Furthermore, this system can also be a reference for studies using images from different cereal grasses.

## Introduction

Stomata have probably received more attention than any other single vegetative structure in plants [1], for they regulate gas exchange between the plant and the environment [2]. Such structures stand for tiny pores on the surfaces of leaves, stems, and parts of angiosperm flowers and fruits [3, 4]. Due to the controlling of the exchange of water vapor and CO^2^ between the interior of the leaf and the atmosphere [3], several plant processes are related to the opening and closing movements of the stomata, such as photosynthesis, transpiration stream, nutrition and metabolism [1, 4]. The control of stomatal aperture requires the coordinated control of multiple cellular processes [3], and its morphogenesis is affected by several environmental stimuli [1–3].

**Fig 1:**
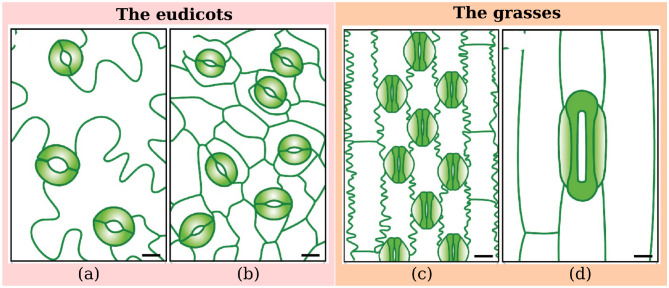
Variation of stomatal traits in terms of size and density from four different plant species: The eudicots are (a) *Arabidopsis thaliana* and (b) *Phaseolus vulgaris*; The grasses are (c) *Oryza sativa* and (d) *Triticum aestivum*. Image adapted from [8].

The number of stomata (stomatal traits) per unit area and their shape vary between species and within species because of the influence of the environmental factors during growth, leaf morphology, and genetic composition. Another characteristic with significant variation concerns the spacing of stomata, which may be relatively evenly spaced throughout a leaf, located in regular rows along the length of a leaf, or they may be clustered in patches [1, 4]. Fig 1 shows four different plant species and their stomatal traits.

Since the types of stomatal configuration are profoundly different, the study and identification of these pores are vital points to understand several mechanisms of plants [5]. Even with such relevance, we still know little about the stomata of cereal grasses [6]. Moreover, the examination of stomata from microscope images is hindered by the manual measurement process, which is highly dependent on biologists with expert knowledge to identify and measure stomatal morphology correctly [7].

In this scenario, to assist the biological community in performing stomata studies, we proposed an automated strategy for stomata detection and classification in microscope images using machine learning through deep and transfer learning techniques. Our work is seminal because it is less time-consuming when examining stomatal behavior, thus enabling biologists to use more information from the images and study a broader range of stomata. In this work, we employed microscope images of maize, representing the most produced and consumed cultivars in the world. As far as we are concerned, we have not observed any similar work concerning maize cultivars.

## Related works

The research of stomata image processing started in the 80’s. Recognized as possible pioneers, Omasa and Onoe [9] proposed a technique for measuring stomata characteristics in grayscale images using Fourier Transform and threshold filters for image processing and segmenting [7]. More recently, Sanyal et al. [10] compared tomato cultivars using several morphological characteristics, including stomata measures. Microscope images of different varieties were obtained using a scanning electron microscope, and the segmentation was performed using a watershed algorithm resulting in one stomata per image, followed by morphological operations (e.g., erosion and dilation) and Sobel kernel filters to remove noise and obtain stomatal boundaries. Using 100 images of tomato cultivars and a multilayer perceptron algorithm, the proposed approach achieved 96.6% of accuracy.

Jian et al. [11] aimed at estimating stomata density using three different regions of *Populus Euphratica* leaves. For image processing purposes, an object-oriented classification method was used with parameters such as scale, compactness, and shape. Such an approach presented high accuracy when compared to human-based count, showing advantages over the traditional method to extract the stoma information. Aiming the constant growth and development of stomata image processing studies, [12] published the “Live Images of Plant Stomata LIPS” database. In other work, [13] presented a semi-automatic stomata region detection approach using ImageJ software [14] and a Clustering-Aided Rapid Training Agent-based algorithm [15].

da Silva Oliveira et al. [16] proposed an approach solely based on morphological operations. Initially, a Gaussian low-pass filter was employed to preprocess the images and remove noise. Further, reconstruction operations (e.g., opening and closing) were applied to highlight stomata regions, which were counted based on background intensity differences. As a result, the work reported recognition rates of around 94.3%.

Laga et al. [17] introduced a supervised method for stomata detection based on morphological and structural features. To fulfill such purpose, 24 microscope images were obtained and filtered by normalization together with a Gaussian filter. The images were manually segmented and the width and height parameters extracted. The authors reported results close to a manual counting approach. Later on, a patent for stomata measurement using Gaussian filtering and morphological operations was registered by [18].

Duarte et al. [19] proposed a method to count stomata in microscope images automatically. Initially, the images were converted from RGB to CieLAB to select the best channel for analysis. Wavelet Spot Detection and morphological operations performed the stomata detection step, with results nearly to 90.6% of recognition accuracy.

Jayakody et al. [7] proposed an automated stomata detection and pore measurement system for grapevines. The approach employed a Cascade Object Detection (COD) algorithm with two main steps: (i) first, the COD classifiers are trained using stoma and non-stoma images, and then (ii) a sliding window over the microscope images was used to identify stomata inside it. After its detection, the pore measurement step was performed using binary segmentation and skeletonization with ellipse fitting, for further estimating pore measurements. The authors reported 91.6% of recognition rate.

As observed, the detection of stomata in microscope images has generally been performed with different morphological operations and segmentation approaches. Although various researches have achieved significant accuracies in the last decade [7, 16, 19], improvements are needed for plant species with more significant stomatal variability. Furthermore, the development of image processing methodologies for automatic stomata detection represents a current challenge with a high potential to boost plant science research on stomatal morphology and its implications.

The use of deep convolutional neural networks has been suggested as a powerful approach for diverse applications on automatically extracting abstract features to be used on prediction [20], replacing the need of defining image descriptors. In several fields of science, the introduction of deep learning techniques has enabled the construction of efficient models in scenarios previously considered as unpredictable [21]. For stomatal research, such use is still embryonic. Incorporating this machinery into stomata segmentation may represent the missing key to developing effective prediction systems, as proposed in this work.

## Materials and methods

**Fig 2:**
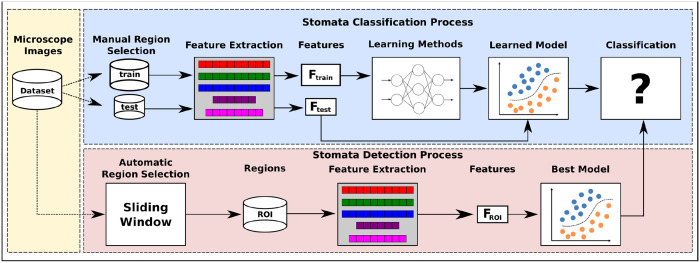
Schematic representation of the proposed pipeline for stomata classification and detection. The proposed approach comprises two main modules: (i) the stomata classification process, where a classification model based on machine learning is created and trained with features extracted from microscope images; and (ii) the stomata detection approach, combining a sliding window mechanism to separate a microscope image into sub-images and a stomata identification process using the model created in (i).

The proposed approach is composed of two different process: (i) stomata detection and further (ii) classification. Fig 2 depicts an overview of the proposed approach.

In the stomata classification process, the first step is to manually collect and label a subset of stomata and non-stomata regions from the microscope images dataset, creating two disjoint sets of subimages, i.e., *train* and *test*. Such sets are subjected to an image descriptor that encodes the visual properties of the subimages into feature vectors (i.e., *F*_*train*_ and *F*_*test*_ for the *train* and *test* sets, respectively). Further, the feature vectors *F*_*train*_ are used as input for a learning method, thus creating a learned model for stomata classification purposes. Finally, each feature vector *F*_*test*_ is then classified by this learned model. In the classification process, different image descriptors and learning methods are evaluated through a *k*-fold cross-validation protocol, and the best model is adopted to detect stomata regions on the next step.

Regarding the stomata detection process, a sliding window is used on each microscope image from the entire dataset to create a set of regions of interest (*ROI*), which are subjected to an image descriptor resulting in the feature vectors (*F*_*ROI*_). Finally, each *F*_*ROI*_ is classified by the best model, i.e., a tuple (learning method + image descriptor) computed in the classification process.

### Stomata classification process

**Fig 3:**
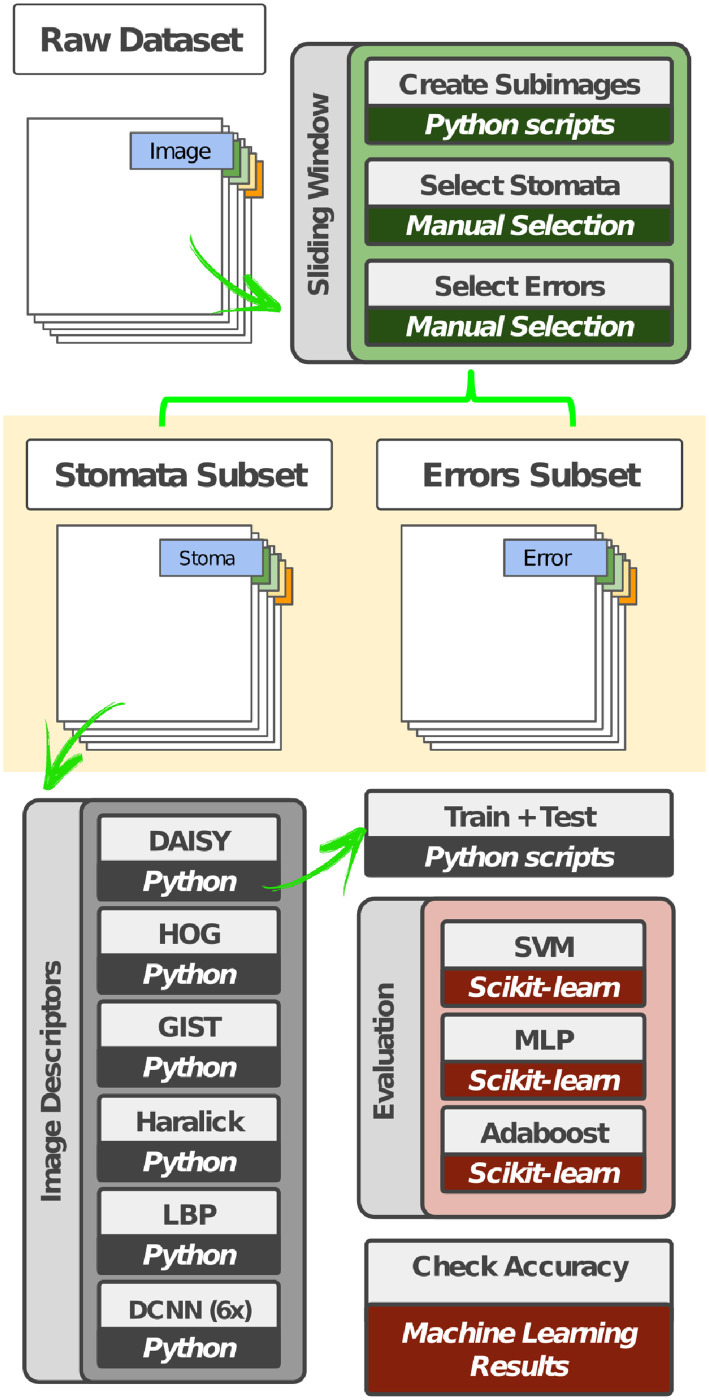
Visual representation of the combination of feature extraction approaches employed and classification algorithms to identify stomata. Based on a manual selection of microscope subimages representing stomata and errors, several image descriptors were employed (DAISY, Oriented Gradient Histogram—HOG, Haralick Texture Features, Local Binary Patterns—LBP, GIST and Deep Convolutional Neural Networks—DCNN) and used to produce features to be used as input to machine learning techniques (Support Vector Machine—SVM, Multilayer Perceptron—MLP, and Adaboost) for identifying stomata.

Fig 3 shows the steps of the stomata classification process proposed in this work. The first step for identifying stomata structures is the manual selection of a set of subimages containing stomata or other plant structures, labeled as non-stomata. Due to the differences between stomata size in distinct microscope images, we adopted a region/window of dimension 151 × 258 pixels. We observed that such size is enough to include all stomata regions from the dataset images. Therefore, a total of 1, 000 subimages of each class (i.e., stomata and non-stomata) were selected to compose the new dataset.

Once the dataset has been created, the next step is to extract visual properties from the subimages using image descriptors and deep convolutional neural networks.

#### Image descriptors

In this work, we evaluated five different image descriptors, being two local descriptors (DAISY and HOG), two texture descriptors (Haralick and LBP), and one shape descriptor (GIST).

- **DAISY**: this descriptor is inspired from SIFT [22] and GLOH [23] descriptors, which relies on gradient orientation histograms. For an input image, orientation maps are calculated based on quantized directions using Gaussian kernels. The final descriptor concerns the values from these convolved maps located on concentric circles centered on a location. The amount of Gaussian smoothing is proportional to the radius of the circles [24].
- **Histogram of Oriented Gradients (HOG)**: Feature descriptor based on the creation of histograms with gradient orientation using their magnitude in specific portions of an image [25]. The local shape information is described by the distribution of gradients in different orientations [26].
- **Haralick Texture Features (Haralick)**: At first, a gray-level co-occurrence matrix is computed considering the relation of each voxel with its neighborhood. Using different statistical measures (e.g., entropy, energy, variance, and correlation), texture properties are encoded from the image into feature vectors [27].
- **Local Binary Patterns (LBP)**: It computes a local representation of texture based on the comparison of each pixel with its neighborhood. A threshold for such comparison is defined, and an output image is produced with the binary to decimal values conversion. Further, a histogram is created as the final descriptor [28].
- **GIST**: The descriptor focuses on the shape of the scene itself, i.e., on the relationship between the outlines of the surfaces and their properties, ignoring the local objects in the scene and their relationships [29]. The approach does not require any form of segmentation and is based on a set of perceptual dimensions (naturalness, openness, roughness, expansion, ruggedness) [26].

#### Deep Convolutional Neural Networks (DCNN)

A typical convolutional network is a fully-connected network where each hidden activation is computed by multiplying the entire input by weights in a given layer [30]. In this technique, a connection between traditional optimization-based schemes and a neural network architecture is considered, where a separable structure is introduced as a reliable support for robust deconvolution against artifacts [20].

Once we do not have available a large scale of images to train a deep learning architecture from scratch, a good alternative is to use the transfer learning [31]. Usually, the networks are pre-trained over ImageNet dataset [32], for further adding other layers according to the target application. The last layer can be used for feature extraction purposes (image descriptor).

In this work, we adopted six different deep convolutional neural networks:

- **DenseNet121** [33]: DenseNet121 architecture contains short connections between the input and the output layers. While state-of-art convolutions network with *L* layers implements *L* direct connections, the DenseNet architecture is implemented using $\frac{L(L+1)}{2}$ connections. Therefore, several advantages are provided, such as reducing the number of parameters used in the model, the reuse of features, and feature propagation.
- **InceptionV3** [34] **& InceptionResNetV2** [35]: The GoogLeNet architecture was introduced as GoogLeNet (Inception V1), later refined as Inception V2, and recently as Inception V3. While Inception modules are conceptually convolutional feature extractors, they empirically appear to be capable of learning richer representations with fewer parameters. A traditional convolutional layer attempts to learn filters in a 3D space, with 2 spatial dimensions (width and height) and a channel dimension. Thus, a single convolution kernel is tasked with simultaneously mapping cross-channel correlations and spatial correlations. Being considered a new version of the Inception architecture, the Inception-ResNetV2 (Inception V4) uses a batch normalization over the usual convolutional layers.
- **MobileNet** [36]: Introduced as an efficient and portable DCNN, MobileNet is developed using streamlined architectures which apply depth-wise separable convolutions. By using only two hyper-parameters, this DCNN allows the model builder to choose the correct model size for each application. All layers are followed by a batch norm and a rectified linear unit (ReLU) activation; one exception occurs in the final fully-connected layer, which has no nonlinearity and feeds the softmax classification layer. In total, this model posses 28 layers.
- **NasNet** [37]: NASNet method relies on the search of suitable convolutional architectures on a dataset of interest. Based on the reinforcement-learning method NAS (Neuron Architecture Search), a controller selects the best models, and it is tuned by evaluating the accuracy of each model generated in a sampling process over time. Here we opted to use the lightweight version NasNetMobile.
- **VGG16** [38]: The VGG network with 16 layers is structured starting with 5 blocks of convolutional layers followed by 3 fully connected layers. Two fully connected layers with 4096 ReLU activated units are then used before the final fully connected softmax layer.

#### Machine learning techniques

Concerning the machine learning techniques, we used three different approaches: (i) Support Vector Machine [39] (SVM), (ii) Multilayer Perceptron [40] (MLP), and (iii) Adaboost [41]. The best tuple (i.e., learning method + image descriptor) are then employed to label the new stomata regions on the next process.

### Stomata detection process

**Fig 4:**
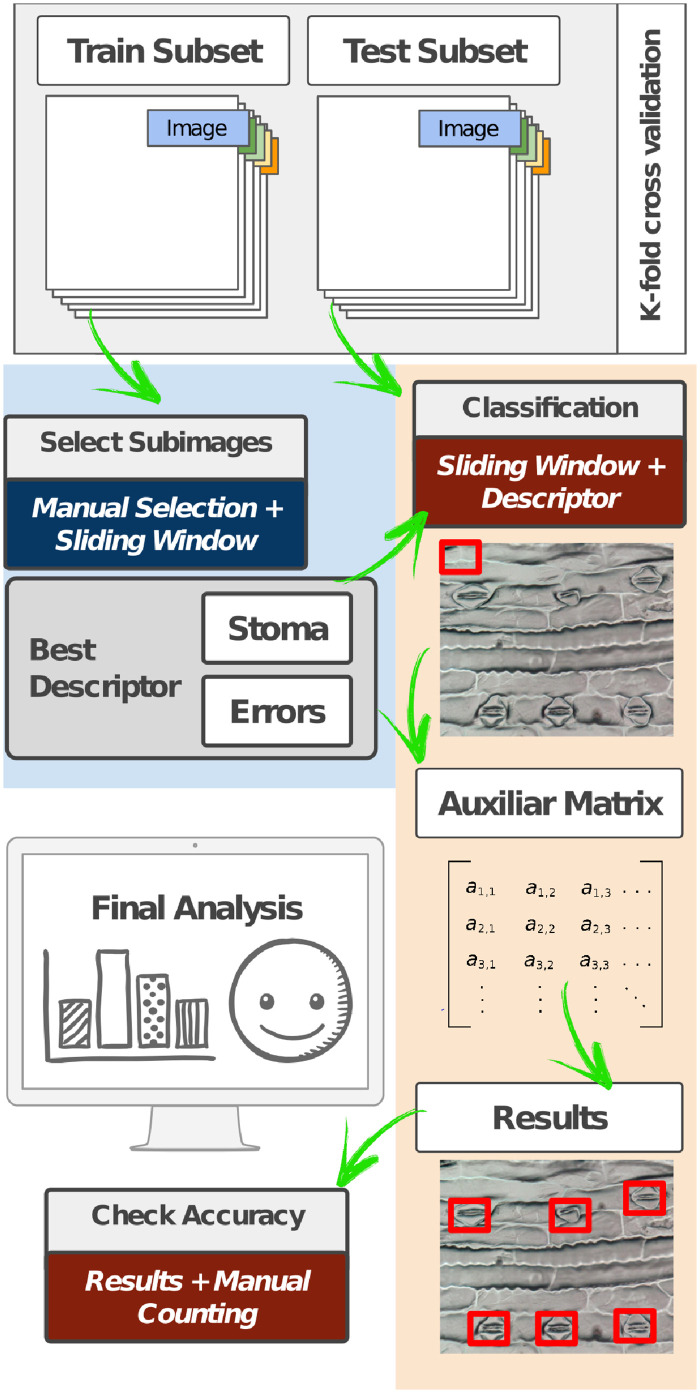
Visual representation of the stoma detection process. From the train and test subsets established according to a k-fold cross validation, a sliding window mechanism was used to go through the image and identify possible regions of pixels corresponding to the stomata.

**Fig 5:**
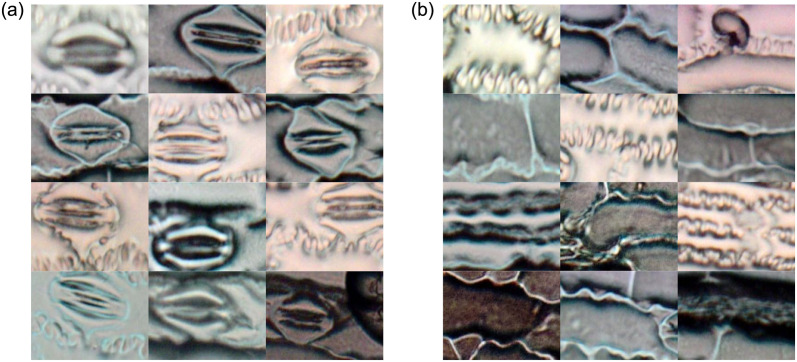
Examples of subimages/regions from the microscope images of maize cultivars corresponding to (a) stomata, and (b) non-stomata. These regions were manually selected and labeled in this work.

Fig 4 depicts the methodology for stomata detection, which is divided into the following steps:

1. **Dataset**: A dataset with stoma and non-stoma subimages (See Fig 5) was created through a manual selection task from microscope images.
2. **Feature extraction**: Once the best descriptor has been found on the stomata classification process, the features of the new dataset are generated and stored into a table with the labels of each category (stoma or non-stoma).
3. **Creation of the learned model**: The descriptors were evaluated using three different learning methods: SVM, MLP, and Adaboost. Based on the best effective results achieved by each learned model (i.e., a tuple composed of a aescriptor + the learning method), the most appropriate learned model is then selected to label the subimage in next step.
4. **Sliding window iteration**: Using a window of 151 × 258 pixels, an iteration over the microscope images is performed, and for each generated subimage, a label (stoma or non-stoma) is obtained using the best-learned model. Due to the possible separation of stoma structures, the windows were created with a stride of 100 pixels in both columns and rows.
5. **Selection of positive regions**: Based on the previous classification, an auxiliary matrix is filled in order to enable the posterior identification of stoma regions. Pixels with a positive occurrence for stoma are separated from the rest of the image, for the further analyzes of such regions.

### Experimental setup

This section describes the image acquisition process, technologies, and evaluation protocol used in this work.

#### Image acquisition

Regarding optical microscope investigation, it has been necessary to separate the epidermis from the remainder of the leaf itself to get a clear view of the cell walls and the shape of the stomata [42]. Herein cyanoacrylate glue was applied to the microscope slide to obtain an impression of the sheet surface to be captured using a camera attached to a microscope. We sampled leaves from 20 *Zea mays* cultivars (maize) granted by Nidera Sementes company (Uberlândia-MG), producing a total of 200 microscope images with different dimensions such as 2, 565 × 3, 583, 2, 675 × 3, 737, and 2, 748 × 3, 840.

**Fig 6:**
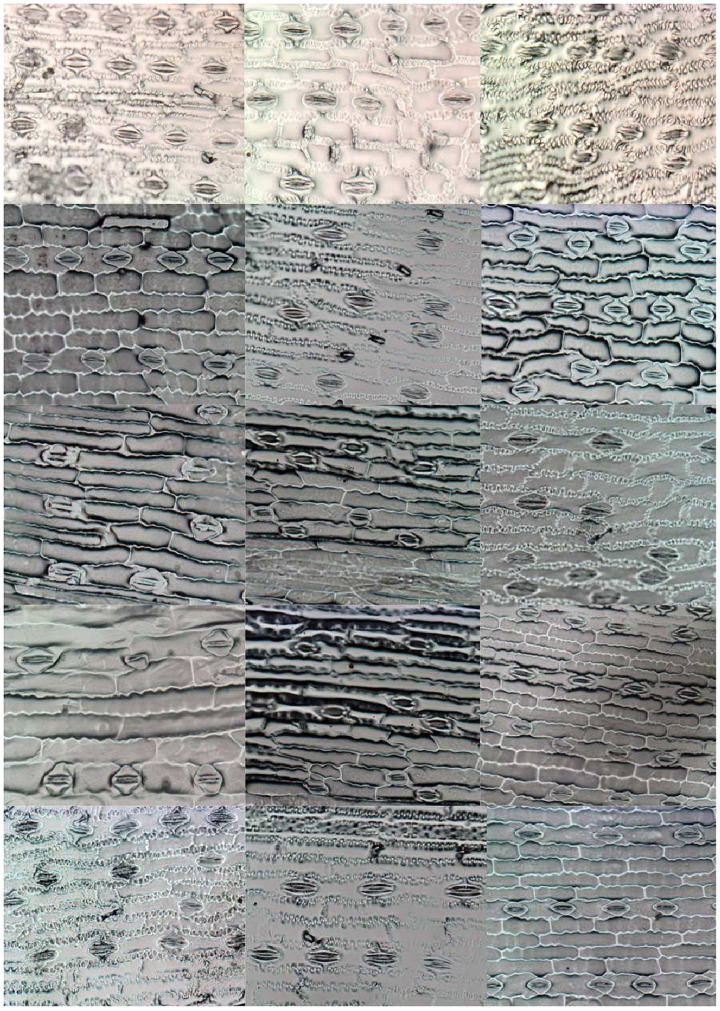
A subset of microscope images used in this work. Each of these images corresponds to different maize cultivars, which show great variability in stoma appearance and configuration.

**Fig 7:**
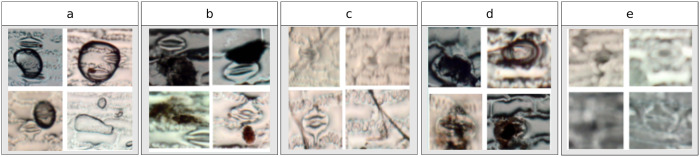
Different types of noise present in the microscopic images: (a) the usage of cyanoacrylate glue can generate air bubbles; (b) the microscope might capture leaves residuals; (c) the leaves might bend and create grooves in the image; (d) degraded stomata due to biological factors; and (e) low image quality due to equipment limitations.

The selected species were treated with colchicine [43] to change their ploidy and cell morphology for further studies. Due to the plant ploidy specificity, different images might have different stomata sizes and width. Besides, as previously mentioned, stomata differentiation is a process that occurs together with the development of plant organs, and herein plants with different ages were used (high intra-class variability), and a clear distinction of the images and plant morphologies can be visualized in Fig 6. In these microscope images, different types of noise and artifacts can be observed as well, as depicted in Fig 7, thus highlighting the challenges faced in this work.

In the experiments, the dataset with 200 microscope images was submitted to the 5-fold cross-validation protocol, i.e., four parts of the dataset compose the training set (160 images), and one part belongs to the test set (40 images). This process is repeated five times. Therefore, in the *stomata classification task*, for each microscope image, 5 stoma and 5 non-stoma regions/sub-images have been manually select to compose training and test sets in an overall of 2, 000 sub-images.

Concerning the *stomata detection task*, respecting the separation of the disjoint sets of the 5-fold cross-validation protocol, each training set created in the stomata classification task is maintained with 1, 600 sub-images. However, the test sets are generated by a sliding window operation. Hence, for each one of the 40 test images, between 876 and 963 regions/sub-images were selected by a sliding window iteration, resulting in approximately 44, 000 sub-images per test set, in an overall of 217, 866 sub-images for the five runs.

#### Programming environment and libraries

All approaches considered in this paper were executed on a personal computer with 2.7GHz Intel Core i7-7500U 2.7GHz Intel Core i7-7500U with 16GB of RAM and NVIDIA GeForce 940MX 4GB graphic card. Similarly, the programming language used in this work was Python2 with the following libraries: scikit-learn [44], pyleargist, scikit-image [45], opencv [46], keras [47] and tensorflow [48]. A considerable part of the libraries was mostly used for feature extraction and deep learning methods purposes.

#### Evaluation protocol

To assess the accuracy of the proposed approach for classifying and identifying stomata regions, we employed a *k*-fold cross-validation with *k* = 5. The classified images represent the test set and the sub-images used to create the learned model were extracted from the training set. A manual count was also performed for each image to evaluate the final results using all windows generated, including the overlapped regions.

## Results and discussion

This section discusses the experiments performed to validate the proposed approach.

### Stomata classification task

In this first experiment, we performed a comparative analysis among five image descriptors (HOG, GIST, DAISY, LBP, and Haralick) and three learning methods (Adaboost, MLP, and SVM) for the stomata classification task. The effectiveness is measured in terms of the mean accuracy considering the 5-fold cross-validation protocol.

As one can observe in Table 1, the best results were achieved by descriptors purely based on gradient information (HOG and DAISY). HOG descriptor with MLP (HOG+ MLP) and DAISY descriptor with Adaboost (DAISY+ Adaboost) achieved 96.0% of mean accuracy. In a comprehensive comparison among all image descriptors, HOG descriptor was the most effective with a mean accuracy of 94.7%, which can be justified by the specific shape of the stoma when compared to other parts. Therefore, this fact can show us that shape is perhaps the most essential visual property for the target application. Although GIST is a shape descriptor, its way of dealing with visual properties globally (holistic) may explain its poor performance in such images.

**Table 1:** Mean accuracies of the classifiers trained with image descriptor features for the stomata classification task. We tested DAISY, Oriented Gradient Histogram—HOG, Haralick Texture Features, Local Binary Patterns—LBP and GIST descriptors, combined with support vector machine, multilayer perceptron and Adaboost machine learning algorithms.

Learning Method	HOG	GIST	DAISY	LBP	Haralick
Adaboost	93.0	79.0	**96.0**	88.0	87.0
MLP	**96.0**	81.0	92.0	85.0	80.0
Linear SVM	**95.0**	81.0	80.0	89.0	86.0
**Mean**	94.7^⋆^	80.3	89.3	87.3	84.3

The values in bold stand for the best descriptor per classifier. Symbol ‘⋆’ denotes the best overall result.

Since deep learning techniques are on the spotlight due to their outstanding results in a number of applications, we also considered them in this work. Table 2 presents the effectiveness results of six different deep learning architectures (DenseNet121—DenseNet, InceptionResNetV2—IResNet, InceptionV3—Inception, MobileNet, NasNet, and VGG16) using three learning methods (Adaboost, MLP, and SVM) concerning the stomata classification task.

**Table 2:** Mean accuracies of the experiments based on deep learning features obtained with the tested convolutional neural network architectures (DenseNet, IResNet, Inception, MobileNet, NasNet, and VGG16) for the stomata classification task.

Classifier	DenseNet	IResNet	Inception	MobileNet	NasNet	VGG16
AdaBoost	95.0	96.0	90.0	96.0	91.0	**99.0**
MLP	98.0	94.0	88.0	98.0	95.0	**100.0**
Linear SVM	80.0	94.0	91.0	98.0	95.0	**100.0**
**Epochs**	10	13	6	7	16	6
**Mean**	91.0	94.7	89.7	97.3	93.7	99.7^⋆^

As one can observe, information based on deep learning features outperformed the handcrafted ones (Table 1), except for HOG descriptor. In this experiment, the classifiers using VGG16 features achieved the best results with 100% of mean accuracy for almost all three learning techniques considered in this work for the stomata classification task.

### Stomata detection task

In this experiment, the classifier based on VGG16 features with Support Vector Machines (SVM+ VGG16) was adopted for the stomata detection task since it obtained the best results in the stomata classification task. Using the sliding window approach to generate possible stomata regions, we have created between 876 and 963 regions/sub-images for each microscope image (overall of 217, 866 sub-images) for the further application of a 5-fold cross-validation protocol.

Table 3 summarizes the effectiveness results considering the classifier SVM+ VGG16. The number of detected stoma regions are compatible with the manual counting, which shows a good performance of the proposed approach. Besides, all folds presented similar effectiveness with around 97.1% of detected stoma regions, i.e., 11, 388 stomata out of the 11, 734 ones present in the dataset. It is also important to clarify that the results achieved in this paper are better than the ones recently reported by Jayakody et al. [7], which obtained an overall accuracy of 91.6% of detected regions.

**Table 3:** Final effectiveness results obtained with the most promising strategy for stomata detection (Support Vector Machine—SVM combined with VGG16 convolutional neural network) based on a 5-fold cross-validation strategy. The performance evaluation considered the number (#) of stomata detected in relation to the real amount.

Fold	# Stoma Manual Counting	# Detected Stoma Regions	Total of Regions	# True Positives	# False Positives
**1**	2,244	2,189 (97.5%)	43,524	5,094	107 (0.02%)
**2**	2,374	2,300 (96.9%)	43,458	5,307	159 (0.03%)
**3**	2,428	2,316 (95.4%)	43,524	5,506	153 (0.03%)
**4**	2,279	2,213 (97.1%)	43,680	5,596	60 (0.01%)
**5**	2,409	2,370 (98.4%)	43,680	5,463	49 (0.01%)
**Mean**	-	2277.6 (97.1%)	-	5,393.2	105.6 (0.02%)
**Overall**	11,734	11388	217866	-	-

**Fig 8:**
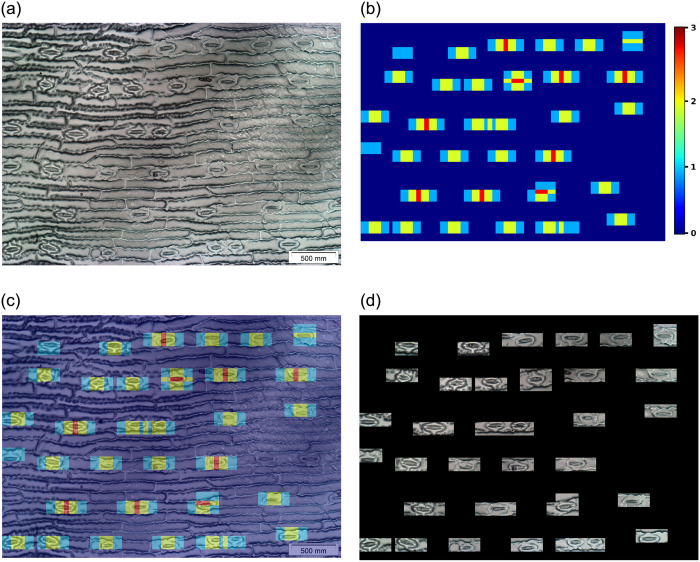
Heatmap representation of the system performance. Based on the sliding window mechanism applied to the original microscope image (a), different regions were considered as containing stomata (b) and used as an image mask (c) for image segmentation (d).

Once the stomata region candidates have been detected in a microscope image (Fig 8(a)), an auxiliary matrix was created to encode the stomata region occurrence (Fig 8(b)), and then a merging between microscope image and auxiliary matrix was performed (Fig 8(c)). Finally, all stomata are identified in the microscope image, as depicted in Fig 8(d).

**Fig 9:**
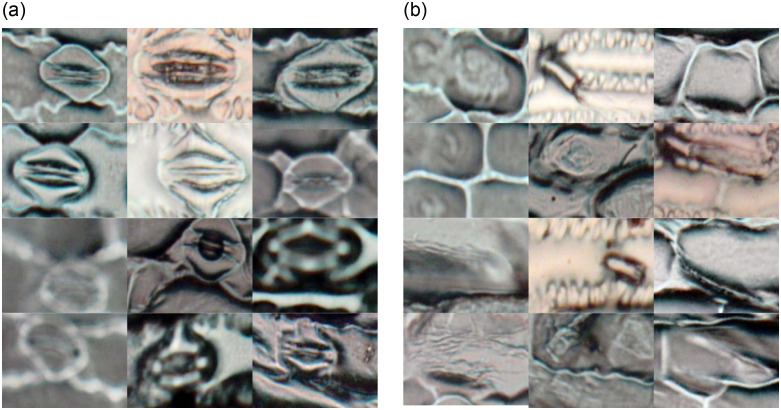
Examples of the stomata classification detection results, including (a) corrected labeled sub-images and (b) false positive results.

We have also analyzed the quality of the effectiveness results. Fig 9 shows the hit and miss-classification results achieved by the proposed system. It is essential to observe that regions/sub-images with low quality have also been correctly classified as containing stoma, as depicted in Fig 9(a). This fact corroborates the usage of the VGG16 features for the stomata detection task. Miss classified regions can be visualized in Fig 9(b). Most of these regions/sub-images represent plant structures that are similar to stomata.

## Conclusion

The understanding of the stomata is of great importance in exploring the evolution and behavior of plants. In this sense, the proposition of computational tools for the detection and classification of stomata is necessary to help better understand these structures and automatically estimate the productivity of the crop. In this work, we proposed a new system for automatic stomata classification and detection in microscope images for maize cultivars based on a transfer learning strategy of different deep convolution neural networks (DCNN).

In the experimental section, we compared the effectiveness of different image descriptors (HOG, GIST, DAISY, LBP, and Haralick) and more complex features based on deep convolutional neural networks (DenseNet, IResNet, Inception, MobileNet, NasNet, and VGG16). We showed that it is possible to obtain stomata classification results (94.7% of mean accuracy) even using well-known image descriptors in the literature, such as the HOG descriptor, which uses less computational power than deep learning architectures. Also, similarly to other applications in the literature, the deep learning architectures extracted the best features from target images. Consequently, they delivered excellent results in both tasks, i.e., classification and detection, achieving 99.7% and 97.1% of recognition rates, respectively. Furthermore, we showed that our system produced robust results in the target tasks even when exposed to a scenario of high intra-class variability of stomata images of maize cultivar. Last but not least, as stomata are responsible for diverse plant biological processes, our findings can significantly benefit future research. As future work, we intend to develop a computational toolkit to support specialists in the biology area in their studies.

## Supporting information

S1 File (TXT)
