# Correction: A stomata classification and detection system in microscope images of maize cultivars

**DOI:** 10.1371/journal.pone.0296551

**Published:** 2024-01-02

**Authors:** Alexandre H. Aono, James S. Nagai, Gabriella da S. M. Dickel, Rafaela C. Marinho, Paulo E. A. M. de Oliveira, João P. Papa, Fabio A. Faria

There are errors in the Funding section. The correct Funding statement is: FAF received support of the Brazilian scientific funding agency CNPq through project #408919/2016-7 and São Paulo Research Foundation FAPESP grant #2018/23908-1. JPP received support of the Brazilian scientific funding agency CNPq through project #307066/2017-7. FAF received GPUs as donation from NVIDIA Corporation for his research. FAF and JPP received support from the Coordination for the Improvement of Higher Education Personnel (CAPES).
